# Region of interest (ROI) selection using vision transformer for automatic analysis using whole slide images

**DOI:** 10.1038/s41598-023-38109-6

**Published:** 2023-07-13

**Authors:** Md Shakhawat Hossain, Galib Muhammad Shahriar, M. M. Mahbubul Syeed, Mohammad Faisal Uddin, Mahady Hasan, Shingla Shivam, Suresh Advani

**Affiliations:** 1 Department of Computer Science and Engineering, Independent University Bangladesh, Dhaka, 1229 Bangladesh; 2 RIoT Research Center, Independent University Bangladesh, Dhaka, 1229 Bangladesh; 3 Department of Pathology, SL Raheja Hospital, Mumbai, 400016 India

**Keywords:** Biomedical engineering, Medical imaging

## Abstract

Selecting regions of interest (ROI) is a common step in medical image analysis across all imaging modalities. An ROI is a subset of an image appropriate for the intended analysis and identified manually by experts. In modern pathology, the analysis involves processing multidimensional and high resolution whole slide image (WSI) tiles automatically with an overwhelming quantity of structural and functional information. Despite recent improvements in computing capacity, analyzing such a plethora of data is challenging but vital to accurate analysis. Automatic ROI detection can significantly reduce the number of pixels to be processed, speed the analysis, improve accuracy and reduce dependency on pathologists. In this paper, we present an ROI detection method for WSI and demonstrated it for human epidermal growth factor receptor 2 (HER2) grading for breast cancer patients. Existing *HER2* grading relies on manual ROI selection, which is tedious, time-consuming and suffers from inter-observer and intra-observer variability. This study found that the *HER2* grade changes with ROI selection. We proposed an ROI detection method using Vision Transformer and investigated the role of image magnification for ROI detection. This method yielded an accuracy of 99% using 20 × WSI and 97% using 10 × WSI for the ROI detection. In the demonstration, the proposed method increased the diagnostic agreement to 99.3% with the clinical scores and reduced the time to 15 seconds for automated *HER2* grading.

A whole slide image (WSI) is a digital image of a pathological specimen generated by a WSI scanner and used in modern pathology for analysis and diagnosis. The WSI scanner converts the entire tissue specimen into a series of image blocks which are then combined to create the WSI. The WSI employs a multi-layer pyramid model, as shown in Fig. 1. The top layer of the pyramid represents the lowest resolution, such as 1 × magnification, which increases with its depth. The bottom layer contains the highest magnification, which provides the highest resolution image. High magnification images such as 20 ×, 40 × or 60 × contain finer details, useful for observing tissue structures and detecting genes, proteins and other biomarkers. However, the processing time increases as the amount of information increases with magnification. Lower magnification images are smaller in dimensions, thus, faster to process. Low magnification images are typically used for analysis that does not require fine details, such as detecting tissue area, abnormalities and artifacts^1^. The WSI can be stored for years without losing quality, shared over a network in minutes for primary diagnosis and secondary consultation and analyzed using computerized methods. The US Food and Drug Administration (FDA) recently approved Philips Ultra-fast WSI scanner for primary diagnosis^2^. As a result, many laboratories are incorporating WSI scanners into their workflow and this adoption of WSI for clinical purposes is arguably one of the most revolutionary technologies introduced to pathology that has the potential to improve the analysis and diagnosis significantly. Several studies that found a high correlation between the WSI-based and glass slide-based diagnosis reported the benefits of incorporating the WSI system into their work^3–5^. NHS Greater Glasgow and Clyde, the largest health board in Scotland and the United Kingdom, has recently begun the complete digitalization of their pathology workflow using the WSI system. In the United States, the largest private cancer center in the world, Memorial Sloan Kettering Cancer Center, has started the adoption process^5^.

**Figure 1:**
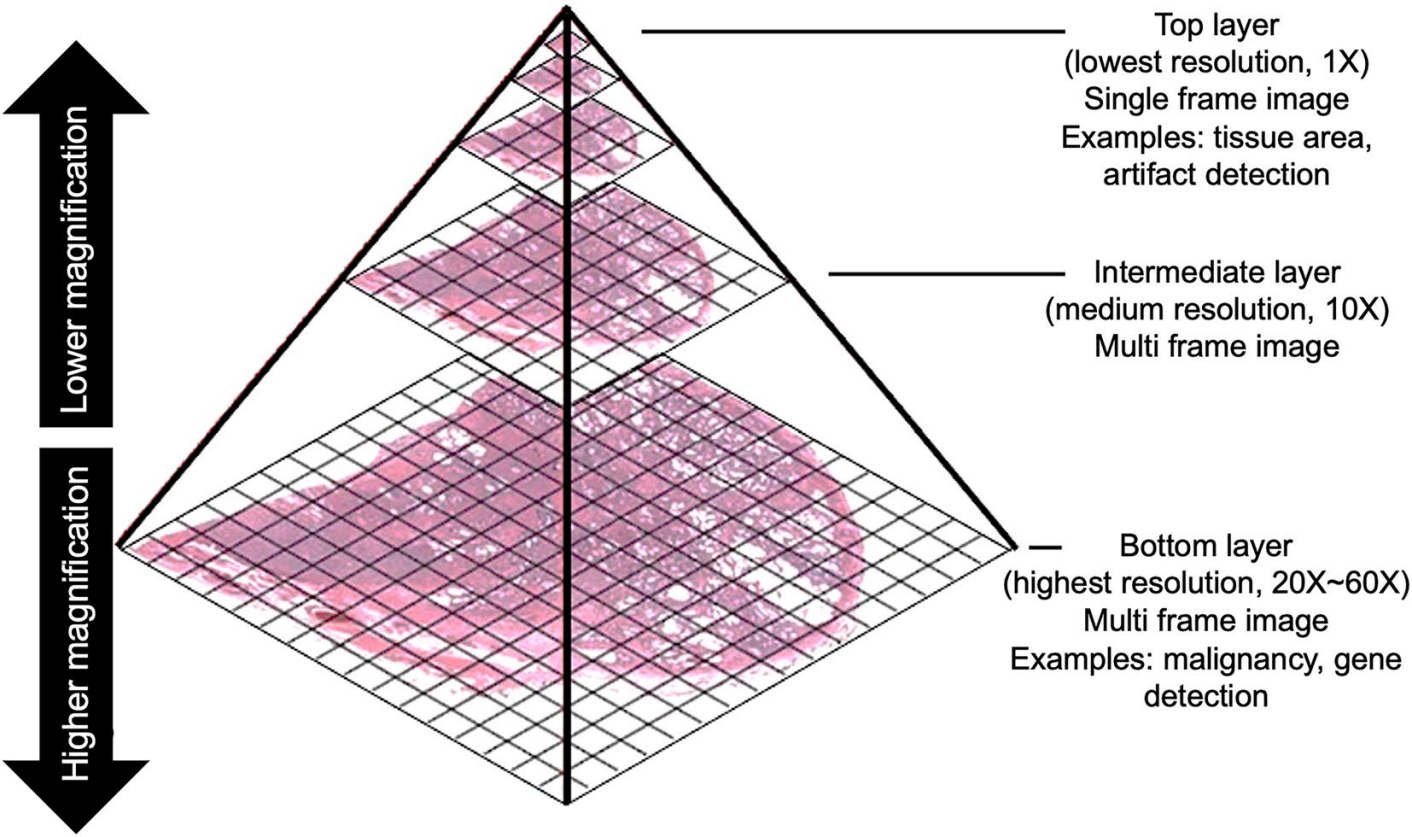
Multi-layer pyramid model of WSI.

The transformation from the microscope to the WSI has enabled the use of computerized algorithms for analysis, overcoming human limitations and minimizing diagnostic errors. This transformation has facilitated the development of artificial intelligence (AI) assisted diagnosis systems, a subcategory of computerized diagnosis systems for morphological and molecular image analysis. The morphological image analysis involves the assessment of tissue architecture and morphology, such as nuclei shape, size and distortion to identify the primary condition of a patient, such as detecting malignant or benign cancer^6^. For the detailed diagnosis of the patient’s condition, molecular analysis is performed, which requires detecting the presence of genes, proteins and other bio-markers such as assessing cancer grade. However, the efficacy of both types of analysis depends on how the algorithm handles the massive WSI data, such as 100,000 × 100,000 pixels. Processing the entire WSI pixel by pixel is inefficient and time-consuming as most of the tissue regions in the WSI are not relevant. Processing irrelevant regions could mislead the computerized algorithms; thus, efficient ROI selection reduces the complexity of training the algorithms. Particularly, molecular image analysis is highly sensitive to region selection as different parts of the specimen show different gene or protein expressions. Fig. 2 shows how different regions of the same specimen show different molecular properties. Table 1 shows how the *HER2* scores change with the region selection, although both regions were cancerous and belonged to the same patient. Therefore, it is necessary to select representative ROIs suitable for the intended image analysis. ROI selection is significant in medical imaging for primary diagnosis, analysis, consultation and training as it is ineffective and often confusing to look at every area of the specimen. Moreover, on many occasions, the analysis is performed using specially stained specimens where tissue structures are missing which is important for identifying the relevant region for diagnosis. Thus, pathologists identify the ROI using the basic H &E specimen, which preserves the tissue structure and then copy the coordinates of ROIs to the special specimen. In digital pathology, deep learning algorithms are used for automated WSI analysis. However, they fail to perform consistently when trained using the entire slide due to diverse histological variances and inconsistencies such as tissue fold, air bubbles, tissue tear, focus blur and excessive color pigment deposit. Thus, machine learning researchers utilize separate methods to detect diagnostically irrelevant areas, which are then processed for analysis. This approach reduces the complexity of deep learning methods for automated analysis, resulting in improved performance in terms of speed and accuracy. Some rely on a semi-automated approach in which an expert manually selects the relevant regions or ROI for automated analysis. However, this approach is not practical and existing automated ROI detection methods fail to achieve sufficient accuracy and reliability. This signifies the need for a separate and reliable ROI detection method. Most computerized image analysis methods rely on the pathologist or an expert to select the ROIs due to the need for efficient and user-friendly automatic ROI detection methods for WSIs. One of the motivations for developing the WSI system was to digitalize pathological workflow to automate image analysis and reduce human intervention considering the worldwide shortage of pathologists^7^. User-dependent ROI selection undermines the goal of automated WSI analysis.

**Figure 2:**
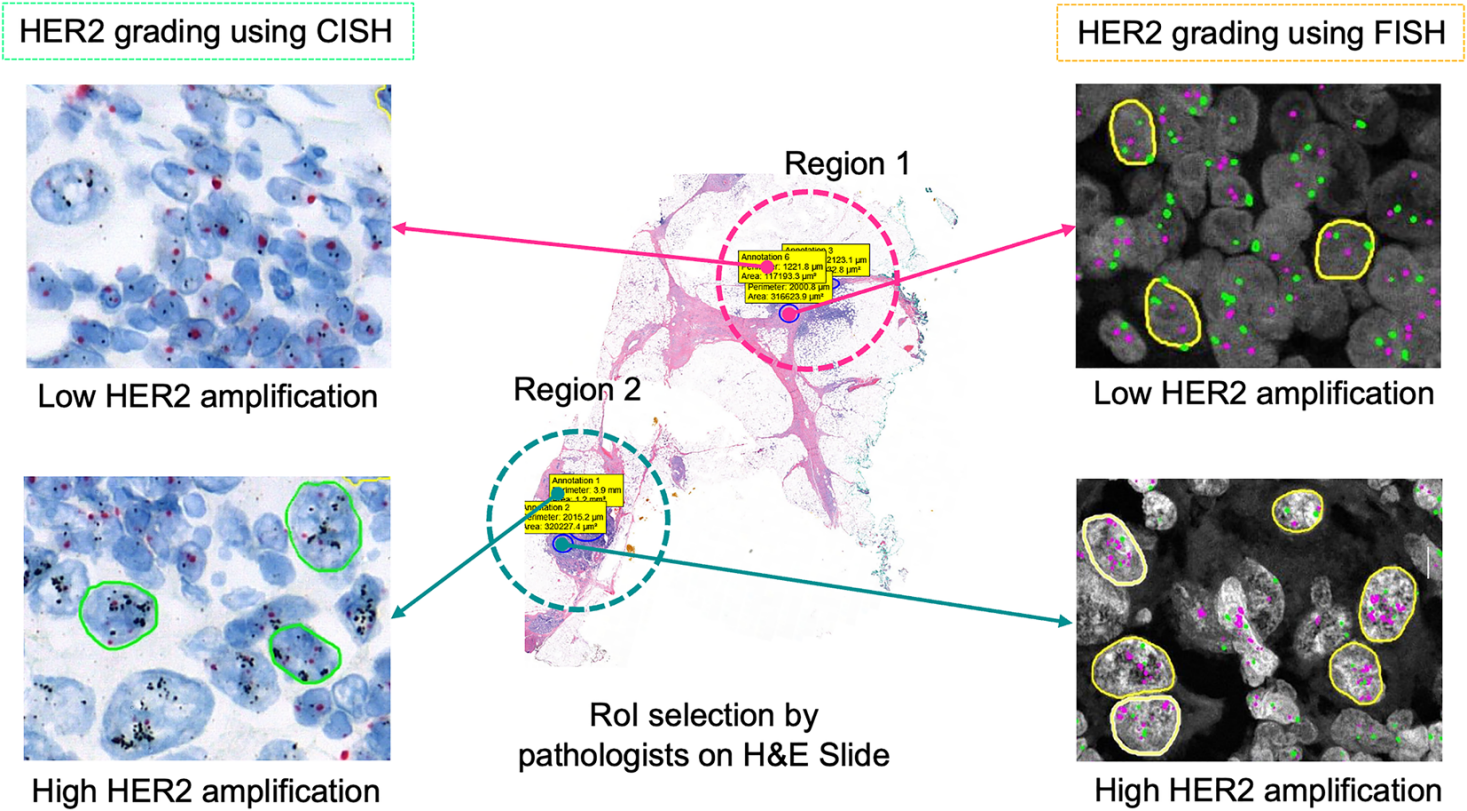
Human epidermal growth factor receptor 2 (*HER2*) status changes depending on the selection of region regardless the test methods.

**Table 1:** Impact of ROI selection on the *HER2* scoring.

Quantified regions	*HER2* score
Only region 1	5.35
Only region 2	1.58
Both region 1 & 2	4.53

Several methods for detecting ROI from WSI have been reported. These methods can be broadly categorized into two groups: (1) methods that utilize image-features^8–12^ and (2) methods that utilize the psycho-physical characteristics of pathologists^13,14^. Image feature-based approaches take advantage of statistical or structural information of tissue such as nucleus shape, texture, color distribution, local binary pattern and others. Pathologists’ activities on the WSI viewer, such as zooming, panning and observation time, are used in the psycho-physical behavioral-based approach to determine the relevance of an ROI. If an ROI was zoomed in and observed for a specific period, it is interpreted as diagnostically relevant. Bahlmann et al. proposed a feature-based method to select diagnostically relevant ROIs from H &E WSI for cancer regions detection^8^. A support vector machine (SVM) classifier was trained using selective statistical features for ROI detection. This approach depended on 40 × images and trained on only a subset of the WSI. As a result, this method produced a large number of false positives when demonstrated on the entire WSI. Romo et al. utilized gray-level texture-based features in combination with color distribution but attained a precision rate of 55%^9^. Barker et al. achieved an accuracy of 93.1% for detecting relevant ROIs for brain tumor detection^10^. This method utilized a modified K-means clustering method but depended on high magnification WSI such as 20 ×. Moreover, the 20 × images were generated by down-sampling the 40 × images, which contain a higher numerical resolution than the actual 20 × images. Therefore, it is difficult to generalize the performance of this method for conventional 20 × WSI. Li et al. proposed a feature-based ROI detection method utilizing low magnification WSI for lung cancer but obtained an accuracy of only 71%^11^. Nugaliyadde et al. relied on the CNN extracted features to improve the accuracy to 91%^12^. Some weakly supervised approaches, particularly based on Multiple Instance Learning (MIL), have been proposed to predict diagnostically significant regions without requiring pixel-level annotation^15,16^. Lu et al. suggested another graph-based weakly supervised approach for diagnosis that allows the use of entire WSI-level data rather than limited ROIs^17,18^. These weakly supervised algorithms show great potential for WSI-based analysis since they require fewer data for training and are computationally faster. However, one major disadvantage of these approaches is that they do not generalize well. As a result, when the scanner profile changes, these methods occasionally fail. The graph-based WSI analysis had a maximum accuracy of 80%, while the MIL-based ROI recognition had an accuracy of 89.74%. The inclusion of irrelevant regions in the analysis could have affected performance since most of the regions in the WSI are not diagnostically relevant.

On the other hand, psycho-physical behavioral-based methods are more functional than feature-based methods; they tend to achieve lower accuracy. Nagarkar et al. proposed a behavioral feature-based method that obtained 100% overlap for only 27% images and 66-99% overlap for 33% images in the demonstration^13^. Mercan et al. proposed another method based on psycho-physical behavior, which determined the diagnostic relevancy of an ROI based on three behavioral features: zoom peaks, slow panning and fixation^14^. This method utilized 40 × WSI to achieve an overlap of 74% with the pathologist’s ROI selection. The behavioral feature-based method extracts features from the view-port analysis in the WSI viewer, such as zooming pattern, observation time and others. The values for these features were then used to infer the pathologists’ interest in ROIs. However, this assumption is not always correct and depends on the pathologists’ intentions. Therefore, in this paper, a more intuitive approach was adopted to confirm the consent of pathologists for ROI selection. Three experienced breast pathologists independently selected the representative ROIs for breast cancer grading. Then, ROIs that they had in common were considered true positives for training the proposed ROI detection method. The rest of the areas of the WSI were considered true negatives. Most of the existing works incorporated the consent of a single pathologist, which made the models biased and thus performed poorly when demonstrated on a heterogeneous dataset. Accuracy and detection time are other important issues. The accuracy of the existing methods is not satisfactory. Moreover, they rely on high magnification images such as 20 × or 40 ×, which is highly time-consuming, thus, not practically feasible. Another major limitation of these methods is that their application was demonstrated only for morphological image analysis and not for molecular analysis, which is more sensitive to ROI selection.

By addressing the shortcomings of current approaches, we present in this study a machine learning-based ROI detection method for automatic image analysis using WSI. This work incorporated the consensus of three expert pathologists for subjective evaluation of the method and yielded an accuracy of over 97% using 10 × images when tested on a heterogeneous dataset. Moreover, this method was demonstrated for an exemplary molecular analysis which is *HER2* grading. Integrating the proposed ROI detection method with the existing *HER2* grading system improved the system’s diagnostic agreement with the clinical *HER2* scores. *HER2* grading is performed routinely for breast cancer patients to plan their treatment and determine suitability for giving Trastuzumab therapy. Fluorescence in situ hybridization (FISH) and chromogenic in situ hybridization (CISH) are FDA-approved gene-based assays for *HER2* grading. In practice, the *HER2* grading process starts by selecting the most representative invasive cancer regions from the H &E slide, which are then copied using the image registration application to the serially sectioned FISH or CISH slide^19^. Then the selected regions are used for counting the *HER2* and CEP17 signals from the nuclei. Finally, the *HER2* to CEP17 ratio is calculated to determine the *HER2* grade, which is highly sensitive to the region selection as illustrated in Fig. 2 and Table 1. Recently, some automated systems were proposed to estimate the grade automatically from FISH or CISH WSI, but they rely on pathologists to manually annotate the ROIs on H &E^5,20,21^. Consequently, it suffers from inter-observer variability, as shown in Table 2. The Mean Square Error (MSE) and the Pearson Correlation Coefficient (PCC) between the users were 4.30 and 0.21, accordingly in terms of *HER2* score for the 12 cases. The Kappa value was -0.15 in terms of *HER2* status. The high MSE, low PCC and negative Kappa values represented high disagreement among the users. Our study found that these methods also suffer intra-observer variability. The proposed ROI detection method was integrated with the automated *HER2* quantification method as an exemplary WSI analysis method to ensure the practical use of the proposed method. We have also investigated the role of WSI magnification and compared CNN and vision transformer models for ROI detection.

**Table 2:** Inter-observer variability for automated HER2 grading due to ROI selection.

Case	User 1:Automated *HER2* score	User 1: automated *HER2* status	User 2: automated *HER2* score	User 2: automated *HER2* status
1	2.50	Pos	2.45	Pos
2	2.45	Pos	2.36	Pos
3	2.22	Pos	3.02	Pos
4	3.83	Pos	6.33	Pos
5	2.14	Pos	1.81	Neg
6	2.64	Pos	1.65	Neg
7	2.57	Pos	1.24	Neg
8	4.26	Pos	1.45	Neg
9	4.15	Pos	2.37	Pos
10	4.24	Pos	6.73	Pos
11	1.30	Neg	4.16	Pos
12	2.65	Pos	6.70	Pos

The major contributions of this study are listed as follows: (1) development of an ROI detection method for WSI analysis, (2) analysis of image magnification in ROI detection, (3) comparison between CNN and Vision transformer models for ROI detection, (4) improvement of computerized *HER2* grading methods in terms of accuracy and time and (5) facilitating the development of a fully automated HER2 grading system using WSI.

## Results

### ROI detection results

The proposed system is designed to detect diagnostically relevant ROIs for automated image analysis using WSI. The selection of ROIs is different for different image analyses. In this study, the proposed system was demonstrated for detecting the representative ROIs for *HER2* grading of breast cancer patients; however, this system can be used for other image analysis applications with necessary adjustments.

**Figure 3:**
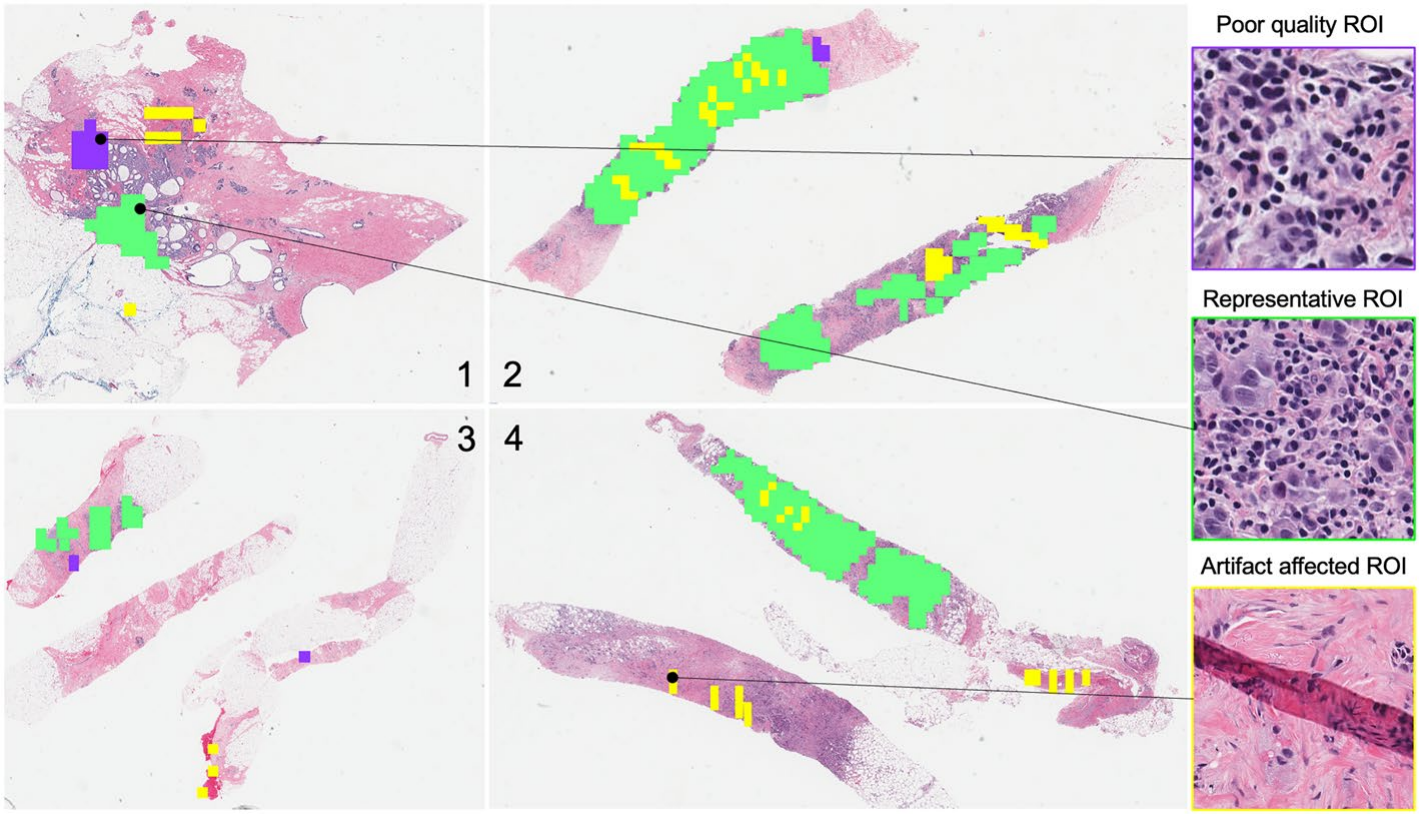
Visualization of ROI detection result produced by proposed method where green, yellow and purple colored-box indicates representative, artifact-affected and poor quality ROIs.

The proposed ROI detection method was trained and tested to classify image blocks as representative or non-representative ROIs from WSI at two different magnifications, 10 × and 20 ×. Though, we recommend 10 × magnification considering its accuracy and speed. The method was tested on 3080 images for each magnification which were not included in the training and validation. An ROI was considered representative or positive in this study if all three expert pathologists selected it as a representative ROI. ROIs chosen by less than three experts or none were deemed negative or non-representative. For the test dataset, the proposed method achieved an accuracy of 97.2% with 100% sensitivity and 94.5% specificity at 10 × magnification. In case 20 × WSI, the accuracy was 100% with 100% sensitivity and specificity. In addition, we demonstrated the proposed method on twelve entire WSIs to ensure its practical use for automated WSI analysis. These WSIs were not used for preparing the training or test dataset. Figure 3 shows the ROI detection results for four WSIs. Prior to ROI detection, the proposed method deploys artifact detection and image quality evaluation. Figue 4 shows the detailed scrutiny of the proposed method for one of the twelve WSI where we identified true positives, false negatives and three types of false positives: (1) selected by two pathologists and the proposed method, (2) selected by one pathologist and proposed method and (3) selected by proposed method but pathologist. In the demonstration using twelve WSIs, the proposed method produced only two false positives and one false negative.

**Figure 4:**
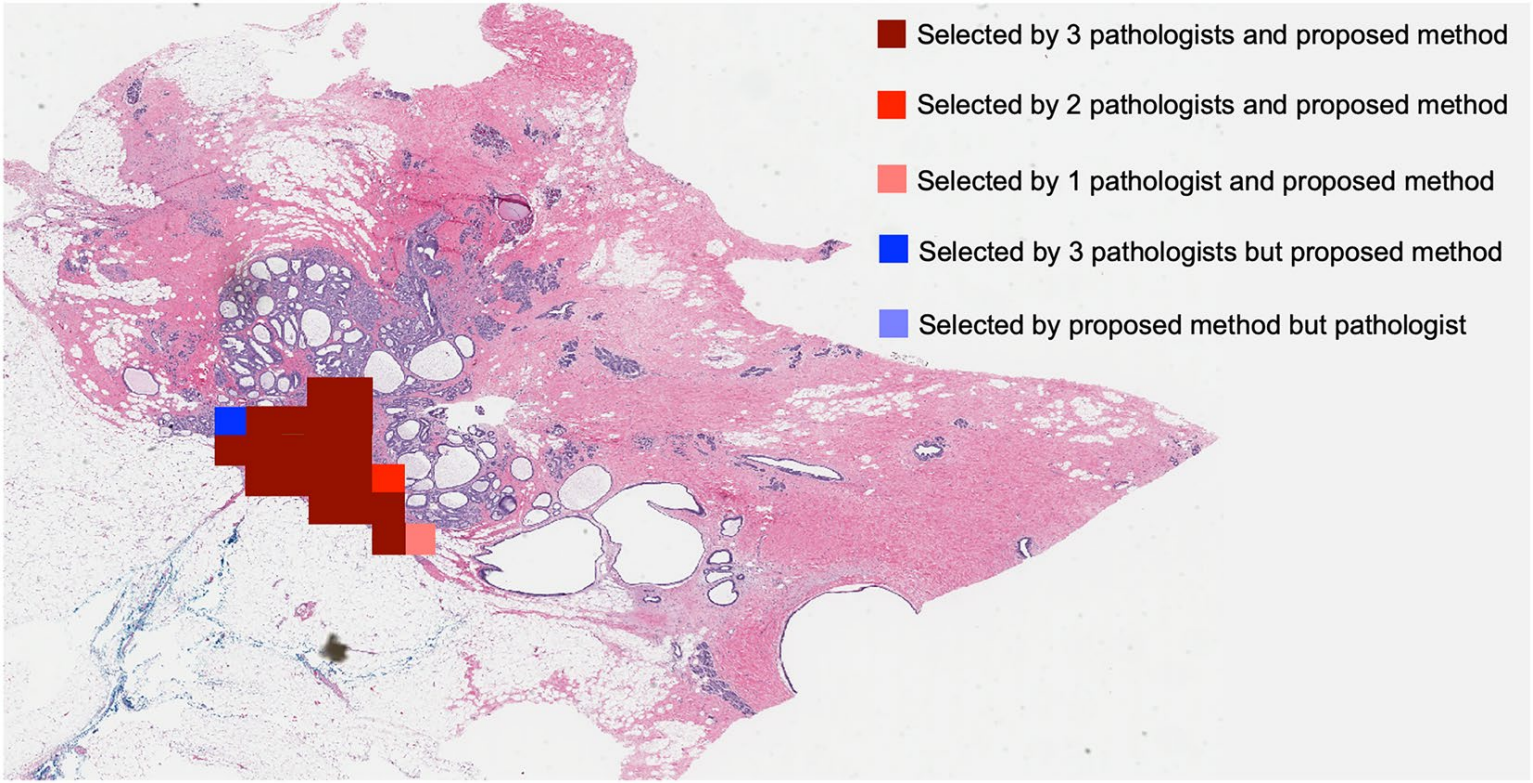
Overlap of ROIs for proposed method and pathologists.

### Comparison between CNN and ViT models

The CNN-based machine learning models are widely used in the medical image analysis task, such as detecting structures and classification tasks. However, the CNNs do not incorporate the position and orientation information for predictions, which are significant for some applications such as ROI detection, where understanding the position of a specific structure in a region is critical. Recently, the vision transformer, primarily proposed for text analysis, has gained immense popularity as an alternative to CNN for image-based applications. CNN processes an image pixel by pixel, whereas ViT divides input images into fixed-size patches to determine the significance of each patch. This approach of transformers has achieved state-of-the-art standard accuracy for numerous computer vision applications. Thus, in this study, we have investigated both approaches to detect the ROIs and compared their performances.

**Figure 5:**
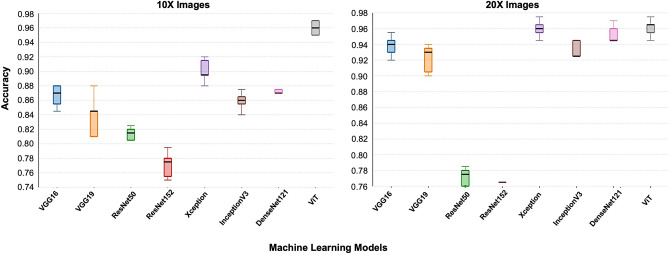
Box-plot of machine learning models for 10 × and 20 × images.

**Figure 6:**
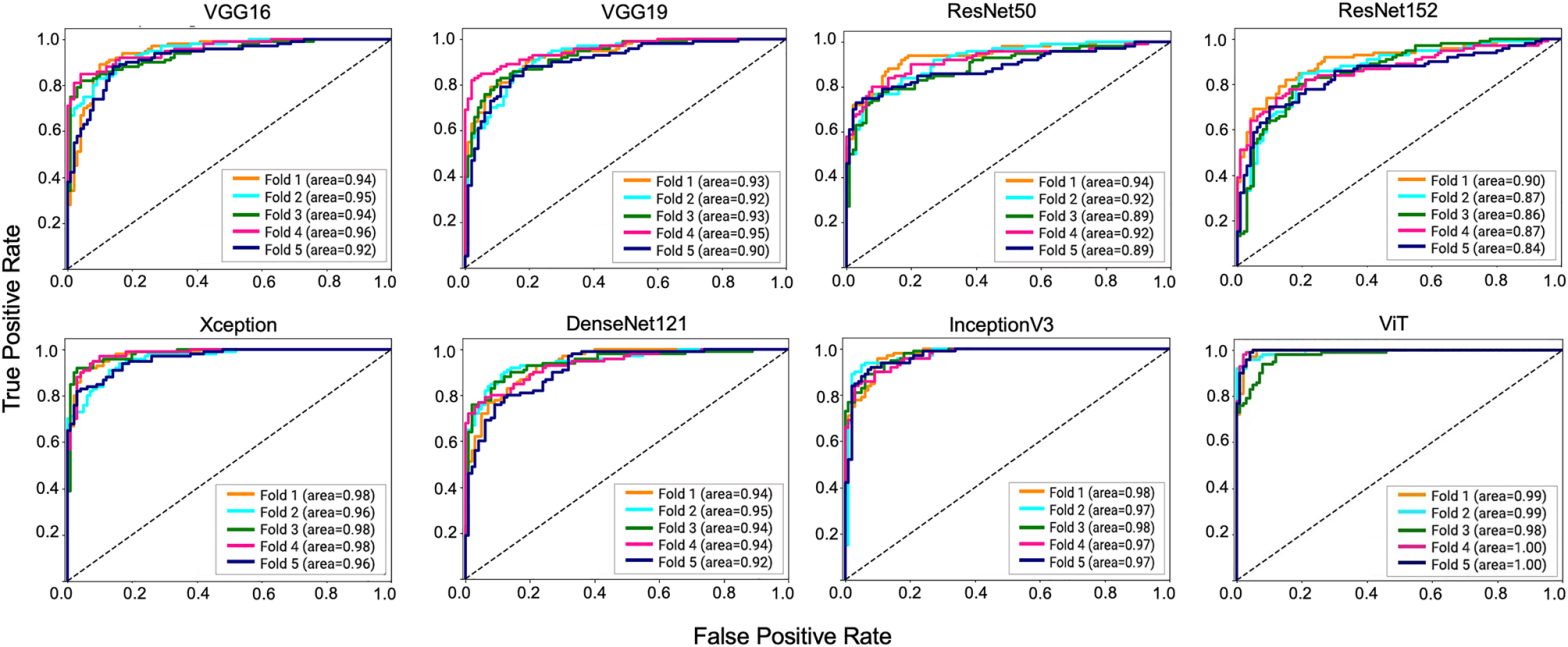
ROC for 5-fold cross validation for 10 × images.

**Figure 7:**
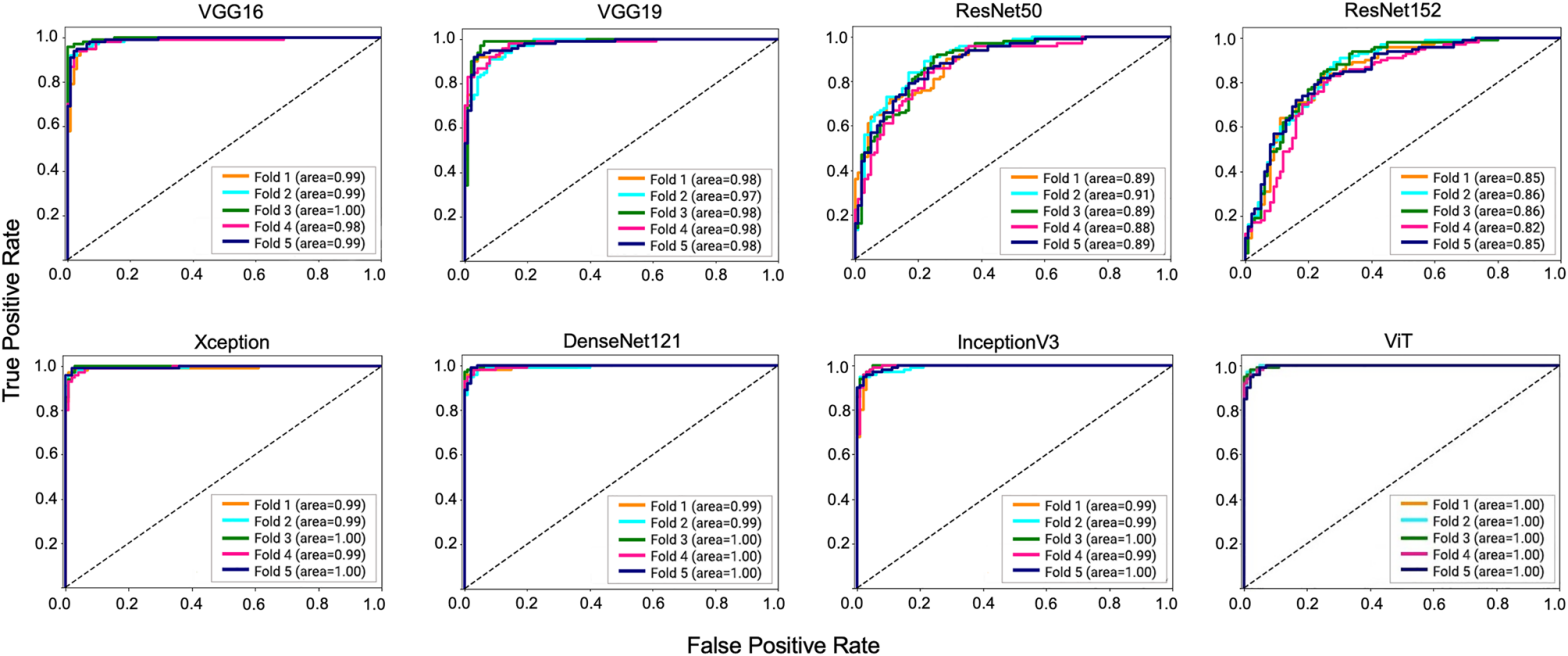
ROC for 5-fold cross validation for 20 × images.

At first, the top candidate networks of seven CNN and ViT models were selected based on their accuracy on the test dataset. The procedure for identifying each model’s top candidate network is explained in Methods Section. Then the top candidate networks of all models were trained and validated in a 5-fold cross-validation experiment. The cross-fold validation experiment was conducted to ensure the robustness and generalized performance of the networks and to identify the best network from the top candidate networks for the proposed method. Then the best networks of each model were trained and validated in a 5-fold cross-validation experiment. For the 5-fold cross-validation experiment, 10,000 images, including 5000 positive and 5000 negative images, were randomly selected from the training and validation dataset. Then these images were assigned to five different groups for the cross-validation experiment. Finally, the models’ average accuracy for 5-folds was calculated and compared. This experiment was conducted using both 10 × and 20 × images. The ViT model achieved the best accuracy for both magnifications, as shown in Table 3. The accuracies of the models were lower using 10 × images compared to the 20 × images. However, such impact of image magnification was the least for the ViT model. The ViT model achieved the highest accuracy of 0.961 and 0.946 among all the models using 20 × and 10 × images, respectively. The sum of differences between the CNN-based models and the ViT were 0.715 and 0.490 for 10 × and 20 × images, respectively. This suggests the ViT performed substantially better on 10 × images than the CNN models. The accuracy of the models was illustrated using a box plot. Figure 5 shows the box plots for 5-fold cross-validation results for 10 × and 20 × images. The ViT had the highest median for both magnifications. The median is the middle quartile of the boxplot that marks the mid-point of the data, as shown by the dark horizontal lines in the boxplots. It indicates that half of the accuracies are greater than or equal to the median and half are less. The boxplots also reveal that the ViT models have a narrow spread of accuracy over cross-validation folds, indicating that they are more consistent than other models. Further, we estimated the area under the curve for the models for the cross-validation experiment, as illustrated in Figs. 6 and 7. The ViT-based models produced the maximum area under the curve close to one in the receiver operating characteristic plot with both magnifications. These results demonstrate that the ViT model is more effective for ROI detection.

**Table 3:** Average validation accuracy of machine learning models in 5-fold cross validation experiments.

Networks	Average accuracy in 5-fold cross validation using 10 × images	Average accuracy in 5-fold cross validation using 20 × images
VGG16	0.866	0.938
VGG19	0.838	0.922
ResNet50	0.806	0.765
ResNet152	0.771	0.763
Xception	0.901	0.960
InceptionV3	0.859	0.940
DenseNet121	0.867	0.947
**ViT**	**0.946**	**0.961**

Significant values are in bold.

### Analysis of image magnification

This study investigated the role of image magnification in ROI detection. The WSI system utilizes a pyramid structure, allowing users to use images of different magnifications. Traditionally, pathologists use low magnification for surfing on the WSI and then confirm an ROI by observing it on the highest magnification, such as 20 × or 40 ×, which is time-consuming. In this study, firstly, the representative ROIs were selected using 40 × magnification by experts and then the machine learning models were trained separately using 10 × and 20 × versions of these ROIs for automatic ROI detection. The models trained on 10 × images were tested for ROI detection using 10 × and 20 × WSI. Similarly, models trained on 20 × images were tested for 10 × and 20 × WSI. Table 4 compares each model’s best networks for the test dataset of two different magnifications. The Table shows that the ViT-based models achieved the best accuracy, sensitivity and specificity in all approaches. When trained and tested on 20 × images, all models outperformed their 10 × counterparts. However, when tested on 10 × images, the accuracy of 20 ×-trained models is much lower than that of 10 ×-trained models tested on 20 × images. This implies that high magnification is preferred for ROI detection; however, it is computationally slower, as shown in Table 5. For all the models, the ROI detection time was roughly lowered in half at 10 × compared to 20 × for the same WSI. The proposed method can identify the representative ROIs from a WSI in less than a minute, whereas manual selection takes 30 to 60 mins.

**Table 4:** Accuracy (ACC), sensitivity (TPR) and specificity (TNR) of machine learning models for test dataset of two different magnification.

Networks	Train: 10 ×, Test: 10 ×			Train: 20 ×, Test: 10 ×			Train: 20 ×, Test: 20 ×			Train: 10 ×, Test:20 ×		
	ACC	TPR	TNR	ACC	TPR	TNR	ACC	TPR	TNR	ACC	TPR	TNR
VGG16	0.892	0.925	0.860	0.603	0.657	0.550	0.996	1.00	0.993	0.564	0.607	0.522
VGG19	0.844	0.883	0.804	0.487	0.498	0.475	0.979	0.998	0.961	0.592	0.659	0.524
ResNet50	0.775	0.834	0.717	0.531	0.540	0.521	0.866	0.925	0.807	0.502	0.545	0.459
ResNet152	0.769	0.802	0.737	0.500	0.540	0.459	0.854	0.889	0.820	0.503	0.546	0.460
Xception	0.847	0.896	0.799	0.535	0.605	0.465	0.990	1.000	0.981	0.767	0.868	0.666
InceptionV3	0.827	0.870	0.777	0.596	0.606	0.587	0.987	0.996	0.979	0.626	0.642	0.611
DenseNet121	0.900	0.870	0.930	0.649	0.622	0.676	0.998	1.000	0.996	0.689	0.696	0.683
**ViT**	**0**.**972**	**1**.**000**	**0**.**945**	**0**.**672**	**0**.**713**	**0**.**630**	**1**.**000**	**1**.**000**	**1**.**000**	**0**.**872**	**0**.**937**	**0**.**807**

Significant values are in bold.

**Table 5:** Time-requirement of machine learning models for two different magnifications of the same WSI.

Networks	Quantified area ($\upmu \hbox {m}^{2}$)	Average time for 10 × (s)	Average time for 20 × (s)
VGG16	23 k	8.50 ± 0.05	15.24 ± 0.07
VGG19	23 k	8.45 ± 0.02	15.31 ± 0.04
ResNet50	23 k	8.66 ± 0.00	15.69 ± 0.05
ResNet152	23 k	9.35 ± 0.04	19.22 ± 0.08
Xception	23 k	8.89 ± 0.01	15.62 ± 0.02
InceptionV3	23 k	9.05 ± 0.04	16.25 ± 0.16
DenseNet121	23 k	9.27 ± 0.07	17.25 ± 1.10
**ViT**	**23 k**	**10**.**33** ± **0**.**12**	**22**.**85** ± **1**.**12**

Significant values are in bold.

### Application to *HER2* grading system

The proposed ROI detection method was demonstrated on an exemplary automated image analysis application which is *HER2* grading using *Shimaris* system^5^. The *Shimaris* was clinically validated for in-house application at Memorial Sloan Kettering Cancer Center, USA. This system determines the *HER2* grade of breast cancer patients from CISH WSIs. At first, the ROIs suitable for determining the *HER2* grade are selected manually by an expert breast pathologist on the H &E specimen. Then, *Shimaris* copies the coordinate locations of the ROIs from H &E to the CISH WSI. After that, it detects the singular nuclei suitable for *HER2* grading from the ROIs using machine learning. Then, it detects the *HER2* and CEP17 biomarkers based on the likelihood of dye concentration. In the next stage, it counts the number of *HER2* and CEP17 biomarkers that fall inside the detected singular nuclei. Finally, it calculates the average *HER2* copy number per nucleus and *HER2* to CEP17 ratio, which are then used to determine the *HER2* grade based on the ASCO/CAP guideline^22^.

The *Shimaris* grading system was chosen in this study since it was designed primarily for completely automated pathological image analysis using WSI. Demonstrating the proposed method for such a system makes it easy to judge its effectiveness while also realizing the system’s benefit. Although *Shimaris* was designed for complete automation, it relied on pathologists for ROI selection which compromises the goal of the system. Initially, this system was limited to bright-field image analysis, such as CISH and later extended to fluorescence imaging, such as FISH. This study reports that the performance of *Shimaris* varies with the ROI selection, which depends on the pathologist as shown in Table 2. Expecting expert pathologists always to be available for ROI selection is unrealistic. Consequently, the system fails when the ROI selection is not appropriate.

In this study, the proposed ROI detection method was integrated with *Shimaris*. Then it quantified twelve systematically selected cases, including ten cases for which the *Shimaris* failed, as listed in Table 6. The integration of the proposed method enabled *Shimaris* to analyze the cases accurately. The PCC between the clinical *HER2* score and *Shimaris* predicted *HER2* score improved to 0.993 when the ROIs for *HER2* quantification were selected using the proposed method. The PCC between the clinical score and *Shimaris* were -0.041 and -0.046, respectively, when the first and second user provided the ROIs for quantification. The MSE between the clinical score and proposed ROI-detection enabled *Shimaris* score was 0.012. The Cohen’s Kappa value was 1, indicating complete agreement between clinical score and proposed ROI enabled *Shimaris*. Integrating the proposed method also improved the turn-round time for *HER2* grading. The time reported earlier for *Shimaris* was 3 to 20 mins, which did not include the ROI selection time. The turn-around for selecting representative ROIs ranges from 30 to 60 mins, which significantly increases the total grading time for *Shimaris*. The proposed method can detect representative ROIs in less than 15 s and on top of that, it reduces the number of ROIs for quantification. A test-retest experiment was performed for the *Shimaris* when integrated with proposed method, as shown in Table 7. The PCC and MSE values between the test and retest scores were 0.998 and 0.004. The Cohen’s Kappa value was 1 in terms of *HER2* status. The low MSE, high PCC and high Cohen’s Kappa value ensured the generalized and consistent performance of the proposed method for selecting representative ROIs. The little variation in the scores was caused by the nuclei and biomarker detection method of *Shimaris*. Thus, it is plausible to conclude that the proposed method increases computerized image analysis applications’ accuracy, efficiency and dependability. It also eliminates the system’s reliance on pathologists, enabling it to be fully automated.

**Table 6:** The proposed ROI selection method improves the correlation between computerized HER2 grades and clinical HER2 grades.

Case	Clinical *HER2* score	Clinical *HER2* status	User 1: automated *HER2* score w/o proposed method	User 1: automated *HER2* status w/o proposed method	User 2: automated *HER2* score w/o proposed method	User 2: automated *HER2* status w/o proposed method	Automated *HER2* score with proposed method	Automated *HER2* status with proposed method
1	0.97	Neg	2.5	Pos	2.45	Pos	1.09	Neg
2	5.50	Pos	2.45	Pos	2.36	Pos	5.20	Pos
3	2.12	Pos	2.22	Pos	3.02	Pos	2.22	Pos
4	2.37	Pos	3.83	Pos	6.33	Pos	2.40	Pos
5	2.17	Pos	2.14	Pos	1.81	Neg	2.14	Pos
6	1.65	Neg	2.64	Pos	1.75	Neg	1.62	Neg
7	1.27	Neg	2.57	Pos	1.24	Neg	1.29	Neg
8	1.45	Neg	4.26	Pos	1.67	Neg	1.56	Neg
9	2.57	Pos	4.15	Pos	2.37	Pos	2.63	Pos
10	1.25	Neg	4.24	Pos	6.73	Pos	1.20	Neg
11	1.20	Neg	1.30	Neg	4.16	Pos	1.17	Neg
12	2.36	Pos	2.65	Pos	6.70	Pos	2.46	Pos

**Table 7:** Comparison between test and retest scores.

Case	Test *HER2* score	Test *HER2* status	Retest *HER2* score	Retest *HER2* status
1	1.09	Neg	1.00	Neg
2	5.20	Pos	5.15	Pos
3	2.22	Pos	2.22	Pos
4	2.40	Neg	2.49	Neg
5	2.14	Neg	2.10	Neg
6	1.62	Neg	1.56	Neg
7	1.29	Neg	1.20	Neg
8	1.56	Neg	1.47	Neg
9	2.63	Pos	2.70	Pos
10	1.20	Neg	1.13	Neg
11	1.17	Neg	1.09	Neg
12	2.46	Pos	2.52	Pos

## Discussion

The overlap between pathologists-selected ROIs and computerized-method processed ROIs is strongly related to the accuracy of automatic image analysis and diagnosis of the computerized method. As a result, medical imaging researchers focus on detecting accurate ROI to design automated image analysis systems. In this paper, we proposed an ROI detection method using a vision transformer for automated image analysis using WSI. This method was trained and tested to detect representative ROIs for *HER2* grading. The experimental results demonstrated that the proposed method could detect ROIs from WSIs for *HER2* grading with acceptable accuracy and precision, even when tested on cases from a different cohort than the one used for training and validation. Whereas some weakly supervised methods avoided pixel-level annotation in favor of analyzing the entire WSI for diagnosis, the proposed method was trained using fine-grained annotation to predict representative ROIs for diagnosis, resulting in higher diagnostic accuracy and consistency.

An essential part of the study was the analysis of image magnification for ROI detection. The proposed method yielded higher accuracy when used with 20 × (1.00) images than with 10 × (0.972), but for 10 × images, the detection time was significantly low. Time is an essential criterion for image analysis systems and we recommend using 10 × magnification in the proposed method. This study found that the patch-wise self-attention mechanism of the vision transformer is highly effective for ROI detection than to pixel-wise feature estimation of CNN. It was also observed that gradually reducing neurons to fit output neurons improves the accuracy of ViT. In the future, the performance of the vision transformer-based approach can be investigated for other related image analysis applications. The proposed method was tested to use with the *HER2* grading system. The proposed method improved the correlation between automated grading and clinical scores, lowered intra- and inter-observer variability and speeded up the analysis.

One major challenge of this study was dealing with WSI color variation. For some WSIs, the hematoxylin stain color was very strong compared to the other WSIs. Non-cancerous regions that are strongly stained appear malignant in such circumstances. This study has a limitation. The proposed method was trained and tested to detect ROIs appropriate for *HER2* grading. However, it is necessary to train and test this method for other image analysis applications. The analysis of image magnification was limited to 10 × and 20 ×. It can be further extended to use 5 × or lower magnification.

One interesting aspect of our work is that the method was demonstrated for molecular image analysis, which is highly sensitive to the ROI selection. Thus, it can be expected that the system will be adaptable for morphological and other molecular image analysis applications. This work is unique in that it incorporated the consensus of multiple pathologists to train and test the model. Independent testing of the method on heterogeneous data ensured that the technique is accurate and robust. Our automated ROI detection method will facilitate the development of a fully automated pathological image analysis pipeline.

## Methods

### Ethics statement

This study was approved under the Institutional Review Board protocol No.02-02-2023 with the Independent University, Bangladesh and all experiments were carried out following approved guidelines. This research used de-identified human data. The IRB committee of Independent University, Bangladesh, waived the need for informed consent for this study as it is impracticable to obtain consent and the research does not infringe the principle of self-determination. Moreover, the research provides significant clinical relevance.

### Data collection and ground truth generation

Three expert breast pathologists annotated 120 H &E WSIs independently to select the most representative ROIs for *HER2* grading. Two different institutions prepared these WSIs, which were digitized by two different scanners. The Yale Pathology electronic database (Yale HER2 cohort) provided 100 of the 120 WSIs, which included 50 *HER2* negative and 50 *HER2* positive cases. Another 20 WSIs were collected from the Cancer Genome Atlas (TCGA HER2 cohort), which included 10 *HER2* negative and 10 *HER2* positive cases. Yale cohort WSIs were produced using Vectra Polaris by Perkin-Elmer scanner using bright field whole slides scanning at 20 × magnification. The TCGA cohort WSIs were generated using an Aperio scanner at 40 × magnification. Following the annotation, the common ROIs between the three pathologists were selected as the representative ROIs for training the machine learning models for automated ROI detection. The remaining ROIs were used as non-representative ROIs for training the models. The ROIs were then exported to png image files at 10 × and 20 × magnifications.

A dataset of 15392 images (224 × 224 pixels) of 20 × magnification was prepared, which included 7696 positives and 7696 negatives for representative ROI detection. Approximately 60% of the dataset was used for training, 20% for validation and 20% to test the models. The training dataset included 4616 positive and 4616 negative images. The validation dataset contained 1540 positive and 1540 negative images. The test dataset also contained 1540 positive and 1540 negative images, which were generated from WSIs that were not included in the training and validation. Another dataset of 15392 images at 10 × magnification was prepared with the same image distribution for training, validation and testing. The 15392 images at 10 × cover a significantly larger area, but we utilize the same number of images at 10 × and 20 × to facilitate model comparison.

### WSI quality evaluation

The WSI quality is occasionally dull, which could be caused by various factors related to the WSI scanner or glass slide specimen. The most typical problems with automated WSI analysis are focus errors and tissue artifacts. Therefore, out-of-focus and artifact-affected regions were detected and eliminated while preparing the dataset. Consequently, the proposed ROI detection method incorporates quality evaluation and artifact detection methods to ensure the system’s resiliency. The proposed method relied on the reference-less quality evaluation method proposed previously by Hossain et al.^23^ to eliminate dull and artifact-affected regions.

### Model training, evaluation and selection

For ROI detection, we trained seven popular CNN and vision transfer models and compared their performance. The CNN models include VGG architecture-based networks^24^ VGG16 and VGG19, deep residual learning-based networks^25^ ResNet50 and ResNet152, depthwise separable convolutions based network^26^ Xception, Inception module based network^27^ InceptionV3 and densely connected layer based network^28^ DenseNet121. These models were trained individually to detect the representative ROI from H &E WSI for *HER2* grading. The CNN models were trained using a transfer learning technique in which the convolution base, which works as the feature extractor, was frozen. Only the top fully-connected (FC) layers responsible for determining the class based on the extracted features were optimized for our training images. In training, the convolution base utilized the pre-trained weights from the ImageNet data^29^ to provide the bottleneck features for the given images as the last activation map before FC layers. Then, the FC layers, which contained three dense layers, including a dropout at the top, were trained using the extracted features of our training images. During the training, different combinations of epochs, batch sizes, learning rates, optimizers and loss functions were explored to find the best network for each model, as shown in Table 8. Each CNN model generated 1152 candidate networks (3 Epochs × 4 Batch sizes × 4 Optimizers × 2 Loss functions × 3 Learning rates × 4 Dropouts). The top candidate network of each model was then selected based on their accuracy on test data using an exhaustive grid search which resulted in 7 networks, one for each CNN model. During the training on the fly data augmentation was also applied by flipping, rotating and zooming the images. Table 4 shows the accuracy of the top candidate networks for all models.

**Table 8:** Parameter values of the hyperparameters explored to find the best network of each CNN based model.

Hyperparameters	Optimization space
Models	[VGG16, VGG19, ResNet50, ResNet152, Xception, InceptionV3, DenseNet121]
Epochs	[25, 50, 75, 100]
Batch sizes	[ 8, 16, 32]
Optimizers	[SGD, Adam, Adamax, RMSProp]
Loss functions	[Categorical cross entropy, Kullback Leibler divergence]
Learning rates	[0.01, 0.001, 0.0001]
Dropouts	[0.5, 0.6, 0.7, 0.8]

**Figure 8:**
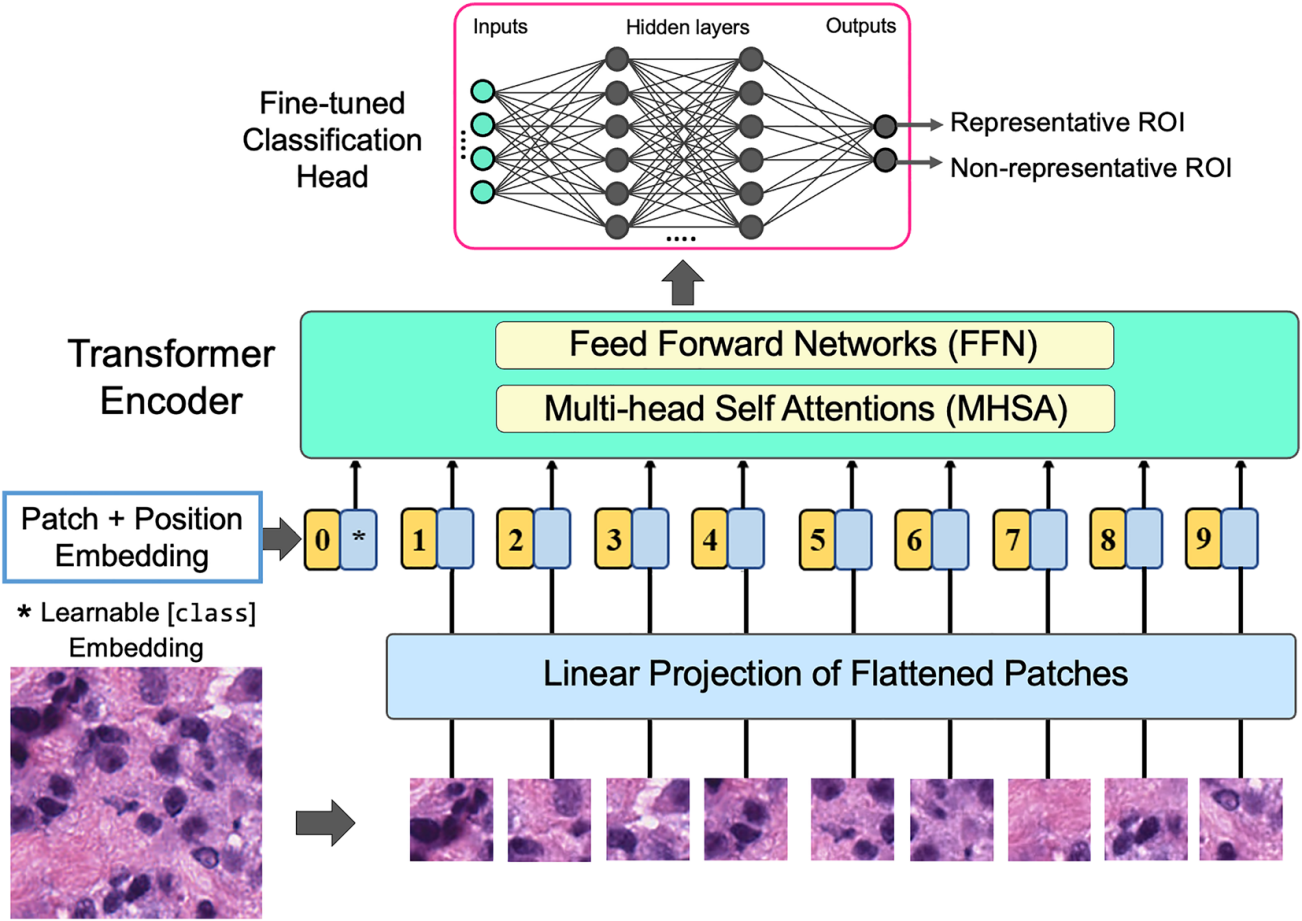
Proposed ViT architecture for ROI detection from WSI.

The convolution operation of CNN models focuses on local information bounded to a small neighborhood of an image. This approach is not always effective, especially for ROI detection, which requires understanding the image’s local and global context. Visual transformers use self-attention techniques that draw information from the entire image, like a global operation^30^. This enables the ViT to understand distant semantic relevance in an image efficiently. ViT has recently gained immense popularity as an alternative to CNN in computer vision. Compared to CNN, ViT shows a generally weaker inductive bias resulting in increased reliance on model regularization or data augmentation when training on smaller datasets. The ViT divides the image into fixed-size patches, which are then flattened and combined with position embeddings to a sequence fed to the transformer encoder. The transformer encoder consists of multiple blocks, each containing normalization, MHSA and multi-layer perceptron layers (MLP). The output of the transformer encoder is fed to a classification head that consists of MLP to map the encoded feature vector to output classes. In this study, the performance of the ViT-based models^30^ was evaluated for ROI detection and then compared with the CNN-based models. We utilized a ViT model pre-trained on the ImageNet dataset and then used our training dataset to train the model in two different approaches: (1) fine-tuned the multi-head self-attention layers (MHSA) of transformer and (2) fine-tuned the MHSA layers and the customized the classification (MCTN) head, as illustrated in Fig. 8.

**Figure 9:**
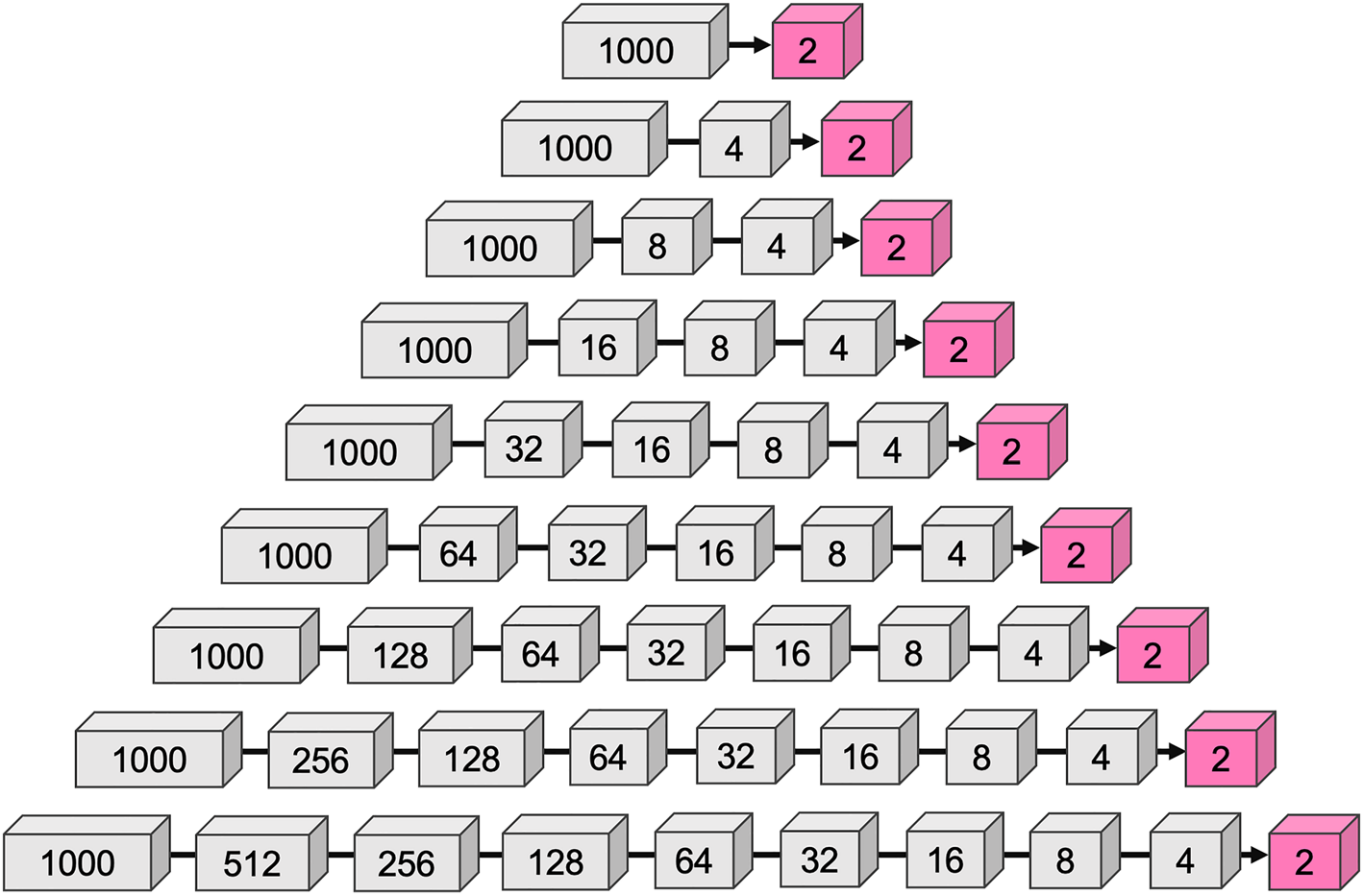
Example of nine different approaches to gradually reduce the neurons to fit two output neurons in the final layer.

In the first approach, the MHSA layers were fine-tuned using our dataset. MHSA allows the network to control the mixing of information between parts of an input sequence, leading to a better representation to increase the model’s performance. The MHSA layers have fewer parameters than the feed-forward network (FFN) layers. Thus, fine-tuning only the MHSA layers without the parameters-heavy FFN layers speed the training time and saves memory. This design was motivated by the study of Hugo et al.^31^, which reported that fine-tuning the weights of the attention layers except the FFN layers is sufficient to adapt vision transformers to other classification tasks. In the second approach, we added more dense layers to gradually reduce the output layers to two neurons. Then fine-tuned, the customized classification head along with the MHSA layers. The ImageNet dataset has 1000 classes; thus, the final layer of the ViT has 1000 output neurons, while the proposed system requires only two output neurons in the final layer. Our experiment found that if the neurons are reduced to 2 directly from 1000, it affects the accuracy. Therefore, the neurons were gradually reduced at different steps. Firstly, the number of neurons was halved at each step which created a set of 9 architectures as illustrated in Fig. 9. The bottom one has 10 layers. Then another 28 approaches were produced from the bottom architecture by skipping only one layer from these 9 architectures. For example, $1000 - 512 - 256 - 128 - 64 - 32 - 16 - 8 - 2$ architecture was produced by skipping the 4-neuron layer. After that, 2 layers were skipped at a time which continued to skip 7 layers at a time. In total, 58 different architectures were produced for the second approach. In both approaches, a classification token was added to the sequence of patch embeddings as a learnable parameter of the model. The classification layers only received the final representation corresponding to this token, the transformer’s output. This represents an aggregate of the patches. Following the linear mapping and the class token integration, standard learnable position encoding associated with the position was added to the model.

In this study, the ViT-B/32 models were used with a 32 × 32 input patch to keep the training time short. The ViT-B/32 is a base variant with 86 million parameters which is the minimum among all variants of ViT. ViT models are also computationally faster with large patches such as 32 × 32. The models were trained for 100 epochs using two different optimizers, AdamW and SGD and a sparse categorical cross-entropy loss function. The model contains 24 pairs of MHSA and FFN. Then, all the CNN and ViT-based networks were compared and the network with the highest test accuracy was selected for the proposed system, which is ViT $(1000-128-64-32-2)$ network derived from the second approach in which both the MHSA layers and the customized classification layers were fine-tuned.

The above experiment was performed for both 10 × and 20 × images. Additionally, the resiliency of the models was tested by applying it to different magnification images. For example, the trained using 10 × images were tested for 20 × images and vice-versa. The time requirements of these models were also estimated for the same WSI. Finally, the model for the proposed method was selected based on its performance on the test dataset. The ViT-based model achieved the highest accuracy, sensitivity and specificity in the test for both 10 × and 20 × image-based experiments. The 10 ×-based ViT achieved 97% accuracy, slightly lower than the 20 ×-based ViT. However, the 10 ×-based ViT is approximately two times faster than its counterpart. Thus, the 10 ×-based ViT model is selected for the ROI detection. Finally, the proposed method was integrated with the *Shimaris* *HER2* grading system and demonstrated for five randomly selected breast cancer patients for *HER2* grading. The proposed ROI selection method increased the correlation of the computerized grading with the clinical results. In the test-retest experiment, the *Shimaris* system produced consistent results when integrated with the proposed method.

### ROI detection from WSI

**Figure 10:**
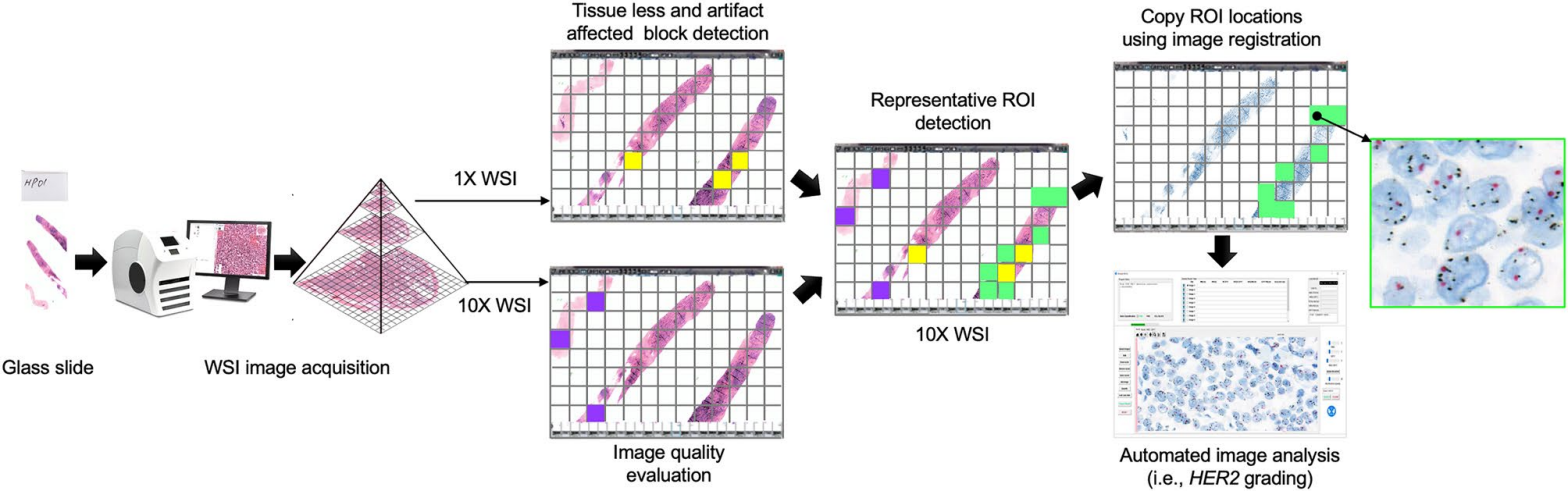
Design of automated WSI analysis using proposed method.

The ROI detection process starts with dividing the entire WSI into fixed-size non-overlapped image blocks of 224 × 224 pixels at 10 × magnification. Then each image block is evaluated for tissue area and artifact using the 1 × version of the corresponding block. If a block contains more than 75% pixels with an intensity value higher than 200, it is rejected as it contains mostly fat or empty area and minimal tissue. A block is also rejected if it contains tissue artifacts. The rest of the blocks are evaluated for image quality to ensure that only good-quality blocks are used for ROI detection. For image quality evaluation, 10 × images are utilized. After that, good quality blocks are classified by the proposed ViT model as representative ROI and non-representative ROI at 10 × magnification. Then, the classification results and their coordinate location are copied to the CISH or FISH WSI from the H &E WSI using an image registration application. Then the detection ROIs are utilized for *HER2* grading or such image analysis. The architecture of automated WSI analysis incorporating the proposed ROI detection method is illustrated in Fig. 10. This design can be used with necessary adjustments for other WSI-based image analysis applications.

## Data Availability

The datasets generated and analyzed during the current study are available in the https://wiki.cancerimagingarchive.net/pages/viewpage.action?pageId=119702524#119702524d22437cd73f54bb3812b2ff952d61a11 Cancer Imaging Archive. The results presented here are in part based upon data generated by the http://cancergenome.nih.gov/ TCGA Research Network.
